# END-OF-LIFE INFORMATION PREFERENCES OF ASIAN OLDER ADULTS: A SYSTEMATIC REVIEW OF THE LITERATURE

**DOI:** 10.1093/geroni/igz038.1536

**Published:** 2019-11-08

**Authors:** Kristina Shiroma, Nathan Davis, Bo Xie

**Affiliations:** The University of Texas at Austin, Austin, Texas, United States

## Abstract

Older adults of Asian ethnic minority groups are often underrepresented in the literature on cultural aspects of end-of-life (EOL) decision making. This literature review aimed to systematically investigate the cultural aspects of EOL decision making for aging adults of Asian ethnic minority groups. In February 2019, systematic searches were conducted in PubMed using MeSH terms “end-of-life”, “decision-making”, and “culture OR cultural”. Articles with human subjects, full text in English, published in the past 10 years, with original, empirical findings were included. After multiple rounds of screening, the final sample included 22 results, with sample sizes ranging from 11 to over 9 millions representing South Asian, Chinese, Korean, Taiwanese, Singaporean, Asian and Asian/Pacific adults. The findings suggest the literature on older Asian adults is present, but limited. Future research is needed to explore cultural aspects of Asian ethnic minority groups in respect to older adult’s information preferences in EOL decision-making.

